# Predictors of diagnostic errors in computed tomography interpretation by emergency physicians leading to changes in clinical management in the emergency department

**DOI:** 10.1007/s10140-025-02357-y

**Published:** 2025-06-23

**Authors:** Naoaki Shibata, Takafumi Yonemitsu, Nozomu Shima, Yuichi Miyake, Tomoya Fukui, Junya Fuchigami, Akira Ikoma, Tetsuo Sonomura, Shigeaki Inoue

**Affiliations:** 1 Department of Emergency and Critical Care Medicine, Wakayama Medical University, Kimiidera, Wakayama, Japan; 2 Department of Radiology, Wakayama Medical University, Kimiidera, Wakayama, Japan

**Keywords:** Emergency department, Computed tomography, Radiological misinterpretation, Emergency physician, Radiologist

## Abstract

**Purpose:**

The use of computed tomography (CT) in the emergency department (ED) has been increasing due to its diagnostic value for emergency physicians (EPs). This study aimed to determine the predictors of EP interpretation errors (IEs) on CT scans leading to change in clinical management (IECM) in both endogenous and exogenous ED visits.

**Methods:**

This single-center, retrospective cohort study included patients with consecutive ED visits initially managed by EPs at our institution over 6 months. Patients who did not undergo CT imaging and presented with cardiopulmonary arrest upon arrival were excluded. CT images were interpreted by emergency radiologists immediately after acquisition, and IEs were identified. The primary outcome was IECM, determined by reference to the clinical management decisions made by EPs. A multivariate analysis was performed to determine the independent predictors of IECM.

**Results:**

Among the 2,037 patients, 158 (8%) had IEs, whereas 52 (3%) had IECM. Multisite CT imaging was the strongest independent predictor for both IECM (OR: 2.25, 95% CI: 1.21–4.19, *P* = 0.011) and IEs (OR: 2.32, 95% CI: 1.61–3.36, *P* < 0.001). Other predictors of IECM were prolonged ED stay and night-time ED visits as clinical factors. Additional predictors of overall IEs were contrast-enhanced CT and abdominopelvic CT as radiological factors.

**Conclusion:**

Multisite CT imaging, which involve multiple organs and extensive diagnostic information, significantly increases the likelihood of misinterpretation, leading to change in clinical management by EPs.

**Graphical Abstract:**

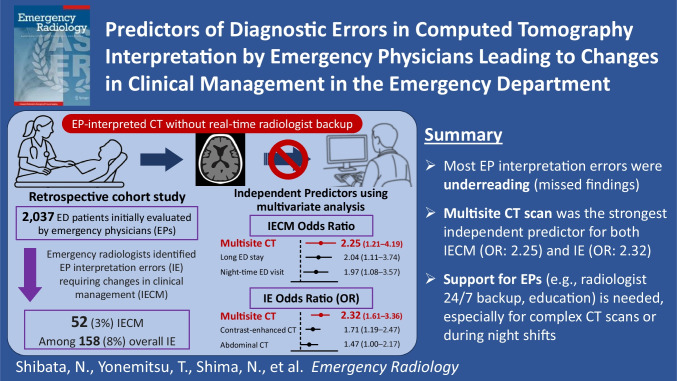

**Supplementary Information:**

The online version contains supplementary material available at 10.1007/s10140-025-02357-y.

## Introduction

The use of computed tomography (CT) in the emergency department (ED) has been steadily increasing due to its high diagnostic value in the initial assessment of emergency care patients [1]. CT imaging enhances diagnostic accuracy for emergency patients with various non-traumatic medical conditions presenting to the ED can significantly influence initial management decisions, including the determination of whether admission is necessary [2, 3]. Additionally, trauma patients who undergo whole-body CT imaging have lower overall mortality rates compared with those who undergo selective CT imaging [4]. Thus, CT utilization in EDs has significantly increased in recent years due to a wide range of visit-related reasons [5], a trend more pronounced than that in non-emergency care settings [6]. In the ED, where quality care must be maintained even during nights and weekends with reduced staffing, 24/7 real-time image interpretation by radiologists ensures the continued utility of CT.

Despite the rapid adoption of high-performance multi-detector CT scanners, radiologists in small community hospitals, particularly in rural areas, face challenges in interpreting images in real-time. Although emergency physicians (EPs) expect radiologists to cover image interpretation beyond their areas of expertise [7], the availability of emergency radiology services, including teleradiology, varies significantly across hospitals [8]. A 2014 survey of after-hours availability for ED image interpretation found that only 27% of United States (US) academic radiology departments have a radiologist on call 24/7 [9]. The shortage of radiologists and the increased workloads caused by increased ED visits often result in EPs interpreting CT images to guide the initial clinical management of patients in the ED [10].

However, the association between CT interpretation errors (IEs) by EPs and subsequent change in clinical decisions remains poorly explored, particularly concerning the use of CT for all emergency conditions, both traumatic and non-traumatic, in the ED. A previous study identified independent predictors for missed injuries in trauma patients undergoing whole-body CT, including multiple traumas involving more than two body parts, age over 30 years, and severe trauma requiring initial resuscitation [11]. By contrast, a comparison of abdominopelvic CT interpretations for acute abdomen between EPs and radiologists found moderate inter-rater reliability (κ = 0.77), indicating that EP interpretation was not associated with patient mortality [12]. Other studies have reported excellent concordance (κ ≥ 0.75) between EPs and radiologists in CT interpretations for both trauma and non-trauma ED patients; however, the association between interpretation discordance and change in clinical management has not yet been evaluated [13]. Moreover, analyses based solely on kappa coefficients provide insufficient insight into the challenges EPs face in clinical practice. Furthermore, previous studies examining CT diagnostic errors in the ED have been constrained by small sample sizes and limited outcomes, failing to fully evaluate the impact of misinterpretation on clinical management.

Therefore, this study aimed to identify the predictors of EP IEs on CT images leading to change in clinical management (IECM) of both endogenous and exogenous ED visits. We hypothesized that radiological factors that increase interpretation complexity (e.g., multisite CT scans) and clinical factors that decrease staffing (e.g., night-time ED visits) are the primary predictors of IECM.

## Methods

### Study design and setting

This single-center, retrospective cohort study was conducted at Wakayama Medical University Hospital between April 1 and September 30, 2019. The study population comprised consecutive patients who presented to the ED of a tertiary-care academic medical center, regardless of the reason for their visit, and were initially evaluated by the EP team. Patients without a CT scan and those who experienced cardiopulmonary arrest upon arrival were excluded. This study was approved by the Research Ethics Committee of Wakayama Medical University. The requirement for informed consent was waived due to the retrospective nature of the study (approval number: 1782).

### Institutional background

Our institution has a tertiary teaching hospital with an advanced emergency center. The EP team initially manages all patients who present urgently to the ED, regardless of the reason for their visit and severity or cause (endogenous or exogenous). The team consists of board-certified EPs, senior residents (SRs), and junior residents (JRs), with the team size varying based on shift schedules. Non-EP specialists provide initial care for acute exacerbations of routine outpatients, perinatal patients, or transfer patients with stable vital signs who have been definitively diagnosed at other hospitals. EPs, SRs, and JRs rotate on two shifts (day shift = 9:01–17:00 and night shift = 17:01–9:00) to ensure continuous 24/7/365 ED coverage. During the day shift, the initial response team includes 2 or 3 EPs, 2 or 3 SRs, and 2 JRs. On the night shift, the team is reduced to a single EP. The ED staffing levels remain the same on weekends and holidays. EPs and SRs collaborate to order imaging studies, including contrast-enhanced CT (CECT). During the study period, CT scans were performed using a General Electric Healthcare 80-slice CT scanner installed in the ED. Consent for CT scans was obtained after explaining the benefits of enhanced diagnostic efficiency and the risks of radiological exposure to the patient or their family. During the study period, no real-time interpretation by radiologists was available. EPs and SRs jointly made decisions based on clinical findings and their own CT interpretations, determining the initial management and ED disposition plan.

### Outcome measures

The primary outcome was the occurrence of an IECM, reflecting the clinical impact of diagnostic errors. The secondary outcome was the total number of IEs made by EPs (IE), representing the radiological aspect.

### Data collection

During the 6-month study period, clinical and radiological data were collected to closely reflect the real-world conditions. Initial clinical data included age, sex, past medical history, underlying chronic disease, transport type (ambulance or emergency helicopter), time of ED visit (day shift = 9:01–17:00; night shift = 17:01–9:00), ED visit on a weekday or weekend/holiday, transfer from a nursing home or other hospitals, initial blood pressure, initial consciousness level, triage assessment using the Japan Triage and Acuity Scale (JTAS), ED crowding, ED length of stay, magnetic resonance imaging, and hospitalization. The Charlson Comorbidity Index was calculated based on the pre-existing conditions provided during patient interviews [14]. Initial systolic blood pressure below 90 mmHg just upon ED arrival was defined as “initial hypotension.” Impairment of consciousness was graded using the Glasgow Coma Scale score upon ED arrival. The JTAS, developed in 2012 based on the Canadian Triage and Acuity Scale (CTAS), is used in most advanced emergency centers in Japan. It includes five levels: level 1 (resuscitation), level 2 (emergent), level 3 (urgent), level 4 (less urgent), and level 5 (non-urgent). A study on JTAS validity found that levels 1 and 2 were associated with significantly more intensive care unit admissions than lower triage levels [15]. Therefore, JTAS levels 1 and 2 were combined as “emergency triage level” for this study. ED crowding was defined as the occurrence of three or more new ED visits within 1 h of each patient’s arrival. Although four major scales were used to assess ED crowding, the National Emergency Department Overcrowding Study is less effective in hospitals with less frequent crowding [16]. As this study was conducted in a non-urban emergency center, the standard ED crowding scale was not used; instead, local criteria based on EP’s subjective assessments of “ED busy (not overcrowded)” were applied. A “long ED stay” was defined as a length of stay exceeding 180 min.

CT images were interpreted by three board-certified radiologists (Na.S., No.S., and T.Y., with 10, 5, and 15 years of experience in emergency diagnostic imaging, respectively), following the standard institutional protocol that required review within 12 h of acquisition, often sooner and including real-time reading for critical cases. The initial CT interpretations and clinical management decisions made by EPs, documented in the electronic medical record, were subsequently reviewed to identify the IEs and IECM. This workflow—involving initial interpretation by the EP team followed by radiologist review within 12 h—represented the standard procedure for ED CT scans at our institution during the study period and had been established 2 years ago when three emergency radiologists were appointed. The standard protocol for trauma whole-body CT included scanning at least three sites (e.g., head, chest, and abdomen) [4]. Accordingly, a “multisite CT scan” was defined as the simultaneous imaging of three or more regions: head, face, neck, chest, abdominopelvic, spine, extremities, and vessels. Additional data on CECT and magnetic resonance imaging were also collected. IEs were categorized as perceptual errors (failure to recognize findings) or cognitive errors (misinterpretation of recognized findings). ED-affiliated radiologists reviewed all CT images to identify underreading as perceptual errors and faulty reasoning as cognitive errors, based on the 12 types of diagnostic imaging errors described in the previous literature [17]. Underreading referred to missed findings, whereas faulty reasoning denoted errors of overreading and misinterpretation, where findings were detected but attributed to incorrect causes [17]. When an IE was identified, discussions were held to determine whether the attending EP would need to change the initial clinical management (e.g., additional medical treatment or informed consent). IECM, the primary outcome, was defined as an IE that necessitated a change in the initial clinical management plan (e.g., additional treatment, specialist consultation, or altered patient disposition). IE, the secondary outcome, was defined based on the framework described by Kim and Mansfield [17]. It encompasses two main categories relevant to acute care: 1) perceptual errors (underreading), where acute findings were missed, and 2) cognitive errors (faulty reasoning), where acute findings were detected but misinterpreted regarding their cause or significance. Patients with multiple IEs were counted once per case. Incidental findings unrelated to acute management, such as fatty liver, asymptomatic inguinal hernia, aortic disease at unrelated sites, or incidentalomas not requiring urgent treatment as oncology emergencies, were excluded from the outcomes. All collected data were entered into a dataset and analyzed statistically.

### Statistical analysis

Continuous variables were expressed as medians with interquartile ranges (IQRs), whereas categorical variables were expressed as numbers with percentages. A multivariate analysis was performed using binomial logistic regression to determine the independent predictors of IE by calculating the adjusted odds ratios (ORs) and 95% confidence intervals (CIs).

Based on previous studies and considering clinical plausibility, seven variables were included in the multivariate logistic regression model. EPs interpreted CT images in environments subject to various clinical disturbances. Therefore, multivariate analysis was performed under the hypothesis that clinical factors affect EP misinterpretations, in contrast to radiologists who interpret images in settings isolated from direct clinical activity. Four variables were included as clinical factors associated with reduced absolute or relative staffing: night-time ED visits, ED busy status, emergency triage level (JTAS 1 and 2), and prolonged ED stay [11, 18, 19]. Three variables were included as radiological factors that increase the complexity of image interpretation: multisite CT scan, CECT, and abdominopelvic CT [11, 13, 20, 21]. Final models were selected using stepwise backward elimination based on Akaike’s Information Criterion. A *P* value of less than 0.05 was considered significant. All statistical analyses were conducted using the JMP software (version 14.1; SAS Institute, Cary, NC, USA).

## Results

### Patient characteristics

**Fig. 1:**
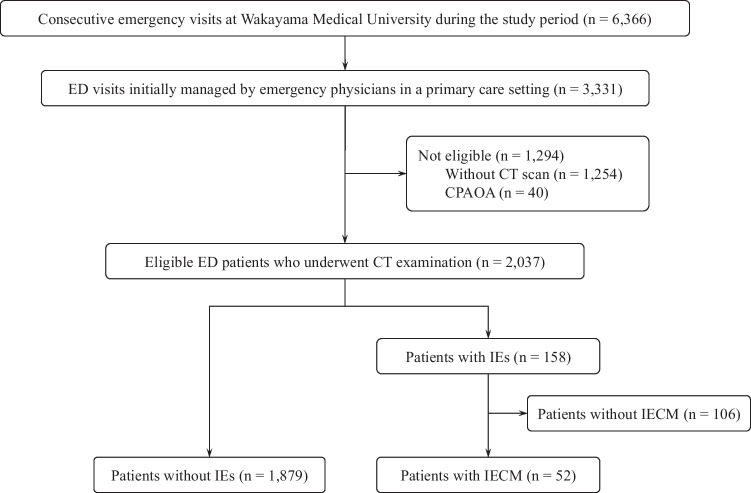
Flowchart of the study process. ED: emergency department, CT: computed tomography, CPAOA: cardiopulmonary arrest on arrival, IE: interpretation error, IECM: interpretation error leading to change in clinical management

**Fig. 2:**
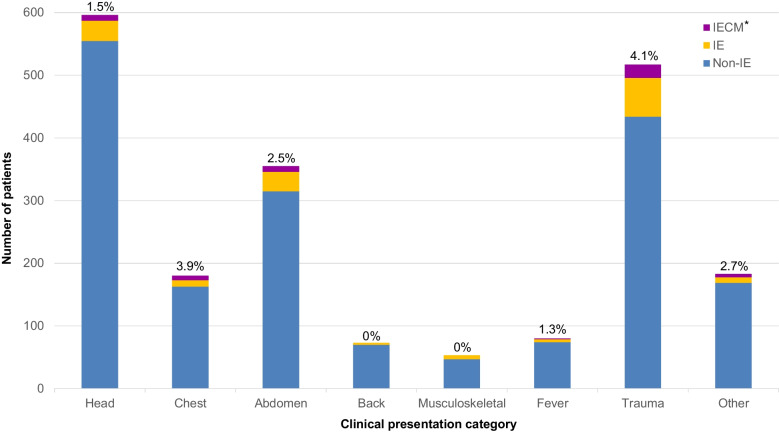
Distribution of interpretation outcomes by reason for ED visit. ED: emergency department, IE: interpretation error, IECM: interpretation error leading to change in clinical management. *Percentages above the top segment represent the IECM rate within each category

During the 6-month study period, 6,366 unscheduled emergency visits occurred at our institution (2,509 by ground ambulance and 245 by air ambulance). Of these, the EP team initially managed 3,331 patients. Among them, 1,207 hospital admissions and 883 trauma patients were registered under the primary care of EPs. A total of 2,037 patients were enrolled and analyzed in the study, after excluding 1,254 patients who did not undergo CT imaging and 40 who experienced cardiopulmonary arrest upon arrival. Among the analyzed patients, IEs occurred in 158 patients (7.8%) and IECM in 52 patients (2.6%) (Fig. 1). The clinical characteristics of the patients are shown in Table 1. Of the 2,037 patients, 1,161 (57.0%) were men, with a median age of 71 years (IQR: 52–81). A total of 1,156 patients (56.8%) visited the ED at night, 819 (40.2%) were triaged as JTAS level 1 or 2, 442 (21.7%) encountered a busy ED, and 881 (43.2%) experienced a prolonged ED stay. Figure 2 illustrates the number of patients by major clinical presentation category, segmented by interpretation outcome (non-IE, IE, and IECM), with IECM rates shown above each bar. Head or neurological symptoms were the most common clinical presentation (*n* = 596, 29.2%), followed by trauma (*n* = 517, 25.4%). Among the 265 admitted trauma patients, the median Injury Severity Score (ISS) was 10 (IQR: 5–19), with 106 patients having an ISS of ≥ 16 during the 6-month study period. Other presentation categories included abdominal symptoms (*n* = 355, 17.4%), chest symptoms (*n* = 180, 8.8%), fever (*n* = 80, 3.9%), back symptoms (*n* = 73, 3.5%), and musculoskeletal symptoms (*n* = 53, 2.6%). Detailed counts for all specific reasons for ED visits, including breakdowns by IE and IECM status, are provided in Supplementary Table S1. The CT scan characteristics, categorized by scan region and scan subtype, are summarized in Table 2. Multisite scans (≥ 3 sites) were performed in 427 patients (21.0%), resulting in overlapping counts across scan regions. The most frequently scanned region was the head (*n* = 1126, 55.3%), followed by the abdomen/pelvis (*n* = 966, 47.4%) and chest (*n* = 852, 41.8%). The use of contrast media was recorded in 427 patients (21.0%). Among the 158 patients with IEs, underreading was identified in 135 (85%) and faulty reasoning in 23 (15%) as IE error types. Among the 52 patients with IECM, underreading was identified in 46 (88%) and faulty reasoning in 6 (12%). The most common sites of IECM were the head in 10 (19%), spine in 9, lungs in 7, abdominal organs excluding the gastrointestinal tract in 7 (2 in the pancreas, 2 in the mesenteric fat,1 in the bile duct, 1 in the spleen, and 1 in the oviduct), gastrointestinal tract in 5 (3 in colon/1 in small intestine/1 in appendix), vascular in 5 (2 in aorta, 2 in the pulmonary artery, and 1 in the peripheral vessels), bony thorax in 3, extremity bones in 3, extremity joints in 2, and subcutaneous soft tissue in 1 patient. In 7 (13%) of the 52 patients with IECM, the IE findings were unrelated to the reason for the ED visit. No IECM was directly linked to mortality during the study period.

**Table 1:** Clinical characteristics of the study patients

Patient characteristics	Overall, *n* = 2037	IE, *n* = 158	IECM, *n* = 52
Age, median [IQR], y	71 [52**–**81]	70.5 [54**–**81]	71 [47**–**81]
Sex, male, *n* (%)	1,161 (57.0)	93 (58.9)	29 (55.8)
Charlson Comorbidity Index, median [IQR]	1 [0**–**2]	1 [0**–**2]	0 [0**–**2]
Ambulance/emergency helicopter, *n* (%)	1,501 (73.7)	125 (79.1)	46 (88.5)
Night-time ED visit, *n* (%)	1,156 (56.8)	89 (56.3)	35 (67.3)
Weekend/holiday ED visit, *n* (%)	830 (40.7)	61 (38.6)	20 (38.5)
Transfer from nursing home, *n* (%)	116 (5.7)	9 (5.7)	4 (7.7)
Transfer from hospital, *n* (%)	346 (17.0)	26 (16.5)	9 (17.3)
Initial GCS score, median [IQR]	15 [14, 15]	15 [14, 15]	15 [14, 15]
Initial hypotension, *n* (%)	126 (6.2)	13 (8.2)	7 (13.5)
Emergency triage level, *n* (%)	819 (40.2)	83 (52.5)	27 (51.9)
ED busy, *n* (%)	442 (21.7)	33 (20.9)	10 (19.2)
Long ED stay, *n* (%)	881 (43.2)	83 (52.5)	34 (65.4)
MRI, *n* (%)	320 (15.7)	18 (11.4)	3 (5.8)
Hospitalization, *n* (%)	1,081 (53.1)	92 (58.2)	27 (51.9)

IQR, interquartile range; ED, emergency department; GCS, Glasgow Coma Scale; MRI, magnetic resonance imaging

**Table 2:** Characteristics of ordered CT scans

CT subtypes	Overall, *n* = 2,037	IE, *n* = 158	IECM, *n* = 52
Scan region			
Head, *n* (%)	1,126 (55.3)	92 (58.2)	27 (51.9)
Facial bones, *n* (%)	196 (9.6)	28 (17.7)	10 (19.2)
Neck, *n* (%)	50 (2.5)	9 (5.7)	3 (5.8)
Chest, *n* (%)	852 (41.8)	93 (58.9)	36 (69.2)
Abdomen/pelvis, *n* (%)	966 (47.4)	104 (65.8)	37 (71.2)
Cervical spine, *n* (%)	288 (14.1)	51 (32.3)	17 (32.7)
Thoracolumbar spine, *n* (%)	151 (7.4)	32 (20.2)	12 (23.1)
Extremity, *n* (%)	129 (6.3)	16 (10.1)	7 (13.5)
Vessel, *n* (%)	104 (5.1)	6 (3.8)	1 (1.9)
Scan subtype			
Multisite scan (≥ 3 sites), *n* (%)	427 (21.0)	65 (41.1)	24 (46.2)
Contrast media use, *n* (%)	427 (21.0)	55 (34.8)	19 (36.5)

CT, computed tomography; IE, interpretation error; IECM, IE leading to changes in clinical management

### Main results

Multivariate logistic regression analysis using stepwise backward selection identified multisite CT scanning as the strongest independent predictor of IECM (OR: 2.25, 95% CI: 1.21–4.19, *P* = 0.011). Prolonged ED stay (OR: 2.04, 95% CI: 1.11–3.74, *P* = 0.021) and night-time ED visit (OR: 1.97, 95% CI: 1.08–3.57, *P* = 0.027) were also significant predictors (Table 3). The independent predictors of IE unrelated to a required change in clinical management were multisite CT scan (OR: 2.32, 95% CI: 1.61–3.36, *P* < 0.001), CECT (OR: 1.71, 95% CI: 1.19–2.47, *P* = 0.004), and abdominopelvic CT (OR: 1.47, 95% CI: 1.00–2.17, *P* = 0.049) (Table 4).

**Table 3:** Multivariate logistic regression analysis of IECM predictors

Variables	Multivariate analysis		Stepwise method	
	OR (95% CI)	*P* value	OR (95% CI)	*P* value
Night-time ED visit	1.96 (1.08**–**3.56)	0.027*	1.97 (1.08**–**3.57)	0.027*
Emergency triage level	1.17 (0.66**–**2.10)	0.589	-	-
ED busy	0.85 (0.42**–**1.73)	0.657	-	-
Long ED stay	2.08 (1.13**–**3.82)	0.018*	2.04 (1.11**–**3.74)	0.021*
Multisite CT scan	2.17 (1.15**–**4.09)	0.017*	2.25 (1.21**–**4.19)	0.011*
Contrast-enhanced CT	1.63 (0.88**–**3.01)	0.119	-	-
Abdominopelvic CT	1.65 (0.83**–**3.28)	0.156	-	-

IECM, interpretation error leading to a change in clinical management; OR, odds ratio; CI, confidence interval; ED, emergency department; CT, computed tomography

*A *P* value of < 0.05 indicating significance

**Table 4:** Multivariate logistic regression analysis of the predictors of total IEs

Variables	Multivariate analysis		Stepwise method	
	OR (95% CI)	*P* value	OR (95% CI)	*P* value
Night-time ED visit	1.13 (0.81**–**1.59)	0.462	-	-
Emergency triage level	1.27 (0.90**–**1.79)	0.175	-	-
ED busy	0.97 (0.65**–**1.46)	0.884	-	-
Long ED stay	1.18 (0.84**–**1.66)	0.348	-	-
Multisite CT scan	2.16 (1.47**–**3.17)	< 0.001*	2.32 (1.61**–**3.36)	< 0.001*
Contrast-enhanced CT	1.64 (1.13**–**2.38)	0.009*	1.71 (1.19**–**2.47)	0.004*
Abdominopelvic CT	1.44 (0.98**–**2.12)	0.066	1.47 (1.00**–**2.17)	0.049*

IE, interpretation error; OR, odds ratio; CI, confidence interval; ED, emergency department; CT, computed tomography

*A *P* value of < 0.05 indicating significance

## Discussion

This study identified multisite CT, prolonged ED stay, and night-time visits as independent predictors of IECM by EPs, whereas multisite CT, CECT, and abdominopelvic CT predicted overall IEs. A key strength of this study lies in evaluating both clinical and radiological factors influencing EP IEs across diverse ED presentations (trauma and non-trauma) in a setting without continuous radiological specialist support.

Nearly 90% of EP CT misinterpretations were classified as underreading (perceptual errors). This contrasts with radiologists, whose errors tend to be more evenly distributed [22]. This finding aligns with the findings of predominant underreading by radiology residents in training, particularly in head and neck CT interpretation [20]. Therefore, although EPs remain vigilant against underreading, they should also be aware that cognitive errors (faulty reasoning) are more likely during thoracoabdominal CT interpretation, which involves greater diagnostic variability and multiple organ systems, compared with head CT. This awareness is essential for optimizing CT utilization.

Multisite CT scanning, the strongest risk factor for both IE and IECM, likely increases the potential for abnormal findings due to the wider scan range. Radiologists are trained to interpret these highly informative images comprehensively and mechanically while integrating clinical context. However, EPs must prioritize findings most relevant to immediate clinical concerns and prompt therapeutic decisions. The growing use of CT in the ED and primary care settings has greatly improved diagnostic accuracy and reliability, resulting in an increasing tendency for EPs to order CT imaging [3, 23]. EPs naturally focus their interpretation on anatomical regions linked to the patient’s presenting symptoms. CECT of the thorax and abdomen (two sites) is valuable in patients with non-traumatic undifferentiated hemodynamic shock, improving diagnostic efficiency and guiding appropriate management [24]. In trauma victims, although some have reported that whole-body CT reduces all-cause and 24-h mortality, others highlight a higher rate of missed injuries in patients with multiple traumas involving two or more sites [11]. Therefore, further validation is warranted to determine whether the diagnostic benefits of multisite CT imaging in both trauma and non-trauma ED patients outweigh the associated rise in IECM risk.

Explaining why prolonged ED stay predicted IECM is challenging due to the limited comparable data. Although previous studies have linked lower triage levels to longer stays [25], our study found no such association, nor any link with ED business. The differences in the definitions of long stay (> 180 min) may partly explain this discrepancy. It is also plausible that patients with vague or complex clinical presentations, leading to diagnostic uncertainty, might inherently require both more extensive imaging (contributing to the association with multisite CT findings) and broader specialist consultations (leading to longer ED stays). Additional factors that may prolong ED stay include delays in definitive treatment, ED bed shortages, preparation time for inpatient wards, and discharge processes. Thus, prolonged ED stay may reflect complex operational dynamics rather than triage level or crowding alone. Further research is needed to disentangle these factors and clarify their relationship with IE risk.

Night-time ED visits predicted IECM but not overall IE. This contrasts with studies indicating stable radiologist interpretation performance for trauma CT interpretation during night shifts [11] and no link between after-hours radiology trainee discrepancies and patient mortality [26]. Although after-hours radiologist errors have been studied, EP performance during night shifts has not. We speculate that the increased IECM risk in our setting may be attributable to reduced staffing and expertise among the EP team during night shifts.

CECT and abdominopelvic CT only predicted IEs, likely due to their higher information content [20] and known high EP-radiologist discrepancy rates, especially for non-traumatic abdominal CT [13]. Previous studies have highlighted the clinical risks, showing that 15% (8/54) of EP misinterpretations of abdominal CT led to missed necessary treatments [12], which underscores the need for caution with EP-only reads. By contrast, our broader study found that 33% (52/158) of overall IEs resulted in IECM. The lack of IECM prediction by these informative CT types may reflect EPs compensating with other clinical skills and diagnostic strategies (e.g., physical exam and ultrasound). Additionally, the substantially lower number of IECM events (*n* = 52) compared with overall IE (*n* = 158) inherently limits the statistical power of the IECM analysis. This may partly explain why factors like CECT and abdominopelvic CT, which were significantly associated with IE, did not emerge as independent predictors of IECM in our analysis model.

ED CT accuracy is closely linked to staffing and workflow. Although increased consultant staffing has been shown to significantly reduce ED length of stay [27], augmenting night-shift staffing remains a challenge for many hospitals. Although the 2000 s saw advocacy for dedicated 24/7 emergency radiologists [28], and continuous onsite coverage has been shown to improve ED flow [29], rising workloads (doubling in the 2010 s relative to ED visit growth [10]) have made purely onsite solutions less feasible. Consequently, teleradiology, despite recognized interpretation discrepancies (approx. 6%) [30], has become the standard for after-hours interpretations in the US, providing a potential solution to prevent EP misinterpretations and facilitate CT use without overburdening radiologists. Continuing radiology education for EPs appears beneficial for improving CT skills, as evidenced by studies on head CT discrepancies between EPs and radiologists [31]. However, formal radiology training remains limited in emergency medicine residencies, despite its recognized importance [32]. Improving the radiologists'work environment and enhancing EP education are crucial for optimizing ED CT utilization, especially since reducing CT orders is challenging [33]. Artificial intelligence may eventually help reduce workloads and errors. However, its clinical application remains nascent, primarily limited to areas like fracture diagnosis [34].

This study has some limitations that require consideration. First, the single-center retrospective study design may have introduced selection bias. Our ED, located in a rural tertiary hospital, may have influenced the results due to the differences in patient severity and crowding compared with urban EDs. Therefore, our results cannot be generalized to hospitals with 24/7/365 image interpretation coverage by on-duty radiologists or teleradiologists. Second, the study was conducted over a six-month period, which may not account for potential seasonal variations in ED visit volume or patient case mix, potentially limiting the generalizability of our findings. Future studies with year-round data could help mitigate this limitation. Third, the acute IECM-associated mortality was not rigorously evaluated as a true outcome. Although radiologist review occurred within 12 h, including real-time reading for critical cases, the exact time delay for each case was not recorded as a variable, and its potential influence on outcomes was not controlled in our analysis. Additionally, potentially fatal IEs requiring acute management changes cannot be overlooked, making it difficult to design IECM studies that accurately evaluate mortality outcomes. Fourth, the difference in CT interpretation accuracy between the attending EPs and SRs was not evaluated.

In conclusion, multisite CT, night-time ED visits, and prolonged ED stays significantly increased EP misinterpretations requiring clinical management changes. A coordinated environment that enables real-time radiologist readings without overload, combined with continuing radiological education for EPs, is essential for effectively utilizing high-performance or whole-body CT in emergency practice, given its increased information content.

## Supplementary Information

Below is the link to the electronic supplementary material.

Supplementary file1 (PDF 82 KB)

## Data Availability

The de-identified datasets used and/or analyzed in the current study are available from the first author upon reasonable request.
