# Supplementary material for: Predictors of diagnostic errors in computed tomography interpretation by emergency physicians leading to changes in clinical management in the emergency department
Source: Emerg Radiol. 2025 Jun 23;32(4):513–22. doi: 10.1007/s10140-025-02357-y (PMC12328500; doi:10.1007/s10140-025-02357-y)
Supplement: Supplementary file 1 — Supplementary file1 (PDF 82 KB) [file 10140_2025_2357_MOESM1_ESM.pdf]

Supplementary Table S1. Reason for ED visit

Reason for visit	Overall, n = 2,037	IE, n = 158	IECM, n = 52
Head/neurological			
Headache, n (%)	72 (3.5)	3 (1.9)	1 (1.9)
Impaired consciousness, n (%)	146 (7.2)	10 (6.3)	3 (5.8)
Transient unconscious, n (%)	40 (2.0)	1 (0.6)	1 (1.9)
Focal signs, n (%)	198 (9.7)	16 (10.1)	2 (3.8)
Vertigo or dizziness, n (%)	63 (3.1)	0	0
Other, n (%)	77 (3.8)	2 (1.3)	2 (3.8)
Chest			
Shortness of breath, n (%)	91 (4.5)	6 (3.8)	3 (5.8)
Chest pain, n (%)	71 (3.5)	4 (2.5)	4 (7.7)
Other, n (%)	18 (0.9)	0	0
Abdomen			
Abdominal pain, n (%)	231 (11.3)	19 (0.9)	3 (5.8)
Hematuria or LUTS, n (%)	11 (0.5)	1 (0.6)	0
Other, n (%)	113 (5.5)	11 (0.5)	6 (11.5)
Back			
Upper back pain, n (%)	39 (1.0)	2 (1.3)	0
Lower back pain, n (%)	34 (1.7)	1 (0.6)	0
Musculoskeletal, n (%)	53 (2.6)	6 (3.8)	0
Fever, n (%)	80 (3.9)	5 (3.2)	1 (1.9)
Trauma, n (%)	517 (25.4)	62 (39.2)	21 (40.4)
Other symptoms, n (%)	183 (8.9)	9 (5.7)	5 (9.6)

ED, emergency department; CT, computed tomography; IE, interpretation error; IECM, IE leading to changes in clinical management; LUTS, lower urinary tract symptoms
